# An investigation on the adsorption and removal performance of a carboxymethylcellulose-based 4-aminophenazone@MWCNT nanocomposite against crystal violet and brilliant green dyes

**DOI:** 10.1039/d2ra07321h

**Published:** 2023-01-31

**Authors:** Raed H. Althomali, Khalid A. Alamry, Mahmoud A. Hussein, R. M. Guedes

**Affiliations:** a Department of Chemistry, Faculty of Science, King Abdulaziz University Jeddah 21589 Saudi Arabia kaalamri@kau.edu.sa mahussein74@yahoo.com maabdo@kau.edu.sa; b Chemistry Department, Faculty of Science, Assiut University Assiut 71516 Egypt; c LAETA-INEGI, DEMec, Mechanical Engineering Department, Faculty of Engineering of University of Porto (FEUP) Rua Dr Roberto Frias s/n 4200-465 Porto Portugal

## Abstract

The multistep chemical modification of carboxymethylcellulose (CMC) in the presence of 4-aminophenazone (A-PH) and multiwall carbon nanotubes (MWCNTs) has been successfully conducted. The environmental performance of this material has been thoroughly investigated. Crystal violet (CV) and brilliant green (BG) were eliminated by utilising a new hybrid nanocomposite material (A-PH-CMC/MWCNTs) from a simulated textile wastewater solution. Using SEM, EDX, XRD and FTIR spectroscopy methods, the detailed characterisation of A-PH-CMC/MWCNT nanocomposites was investigated. The results indicated that the adsorption capacity was dependent on six factors (*e.g.*, contact duration, starting concentration, adsorbent mass, the effect of the solution pH, temperature and the effect of KNO_3_). In addition, thermodynamic and regeneration studies have been reported. According to the theories of pseudo-second-order kinetics, the removal process involves chemical adsorption. The experimental results were best suited by the Langmuir model, in which maximum adsorption capacities of 20.83 and 22.42 mg g^−1^ were predicted for the BG and CV dyes, respectively. The research is a preliminary case study demonstrating the excellent potential of A-PH-CMC/MWCNT nanocomposites as a material for CV and BG dye removal.

## Abstract 2

The adsorption and removal performance of CMC-based 4-aminophenazone and doped MWCNTs NC was investigated CV and BG dyes. The results demonstrate the excellent potential of A-PH-CMC/MWCNTs nanocomposites as a material for CV and BG dye removal.

## Graphical abstract

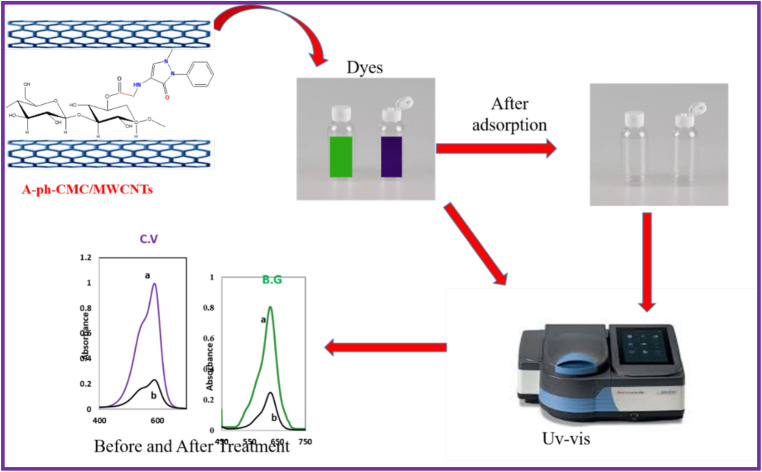

## Introduction

The dye concentration in water has recently increased due to point source contamination, such as the discharge of home and industrial wastewater and many chemical fertilisers in agricultural systems. Pollution makes it difficult for the planet to maintain environmental sustainability.^1^ There is presently around 10–12% of dyes, such as rhodamine B, victoria blue, rose bengal, indigo red, carmine, red 120, eriochrome, methylene blue (MB), black-T (EBT) and thymol blue, according to several studies.^2–4^ Even in meagre quantities (1 ppm), dyes are vividly visible in water and can contaminate aquatic habitats.^5–7^ According to the World Bank, around 17–20% of water contamination is attributable to the dyeing and textile finishing industries. The identified principal wastewater pollutants, including biodegradation,^8^ catalytic photodegradation,^9,10^ electrochemical process,^11^ membrane technology and flocculation/coagulation,^12,13^ were only some of the techniques used. These methods are time-consuming, and some produce sludge as a by-product. Due to its simplicity and cheap cost, adsorption was determined to be an appropriate approach to remove these contaminants. The effluent is treated using an adsorption technique because one of the most likely ways to treat effluents involves a physical or chemical interaction between the surface of a porous material and particles from the liquid phase.^14^ However, the textile, plastic, rubber, cosmetics, pharmaceutical and food sectors currently utilise more than 10 000 commercially available colours.^15^ Moreover, various organic contaminants (*e.g.*, dyes, medicines, pesticides) are increasing in the water due to increased industry, urbanisation and unrestricted human activities. These substances are mutagenic and cause allergic dermatitis, skin burns and serious eye damage.^16,17^ These organic pollutants can affect human and animal health due to their carcinogenicity and poor biodegradability in nature. Some of them are persistent organic pollutants, resistant to environmental deterioration, and continue to enter the ecosystem.^18^ Dye levels in wastewater, for instance, are significantly increasing due to extensive clothing usage and everyday life items. This is one of the greatest problems worldwide.^19,20^ Polysaccharides, a broad class of carbohydrate polymers derived from natural sources, have been distinguished in wastewater treatment applications. This is because of their exceptional properties, including adsorbent performance, renewability, biodegradability, biocompatibility and ease of modification. Nonetheless, polysaccharides have intrinsic drawbacks. For instance, most polysaccharides have low activity and are unsuitable for direct use as adsorbents due to their difficulties in regeneration and reuse. The mechanical characteristics of polysaccharide matrices are often poor and necessitate additional matrices.^21^ Scientists are committed to creating enhanced, multifunctional polysaccharide-based adsorbents that can adapt to harsh environments and have more desired features. Adjusting the physicochemical parameters of polysaccharides may often improve the efficacy of polysaccharide-based adsorbents for wastewater treatment. For instance, polysaccharide-based adsorbents created by cross-linking reactions are the most commonly used method. Carboxymethyl cellulose (CMC) is an anionic cellulose ether with greater water solubility than cellulose. Due to its hydrophilicity, biodegradability and biocompatibility, CMC is often employed in food packaging, sewage treatment and medicine administration.^22–24^ Furthermore, CMC has a carboxyl group (COO) on its side chain, resulting in pH sensitivity. Common modification techniques concentrate on the hydroxyl and carboxyl groups on the CMC molecular chain as reactive sites. Furthermore, using the negatively charged properties of the CMC molecular chain enables its production. It has higher activity through its association with materials with high adsorption properties, and is simultaneously biocompatible, biodegradable and highly adsorbable.^25–28^ In this study, the CMC amide derivative involves converting carboxylic acid to an alkyl ester, and it has been synthesised successfully. First, the carboxylic acid function is converted to an ester *via* Fisher esterification, which requires acidic conditions. Even though the reaction is normally achieved in strong acids (*e.g.*, sulphuric acid), esters have been produced in autocatalytic conditions. Esters are commonly produced by suspending H-CMC in an alcohol reagent combination to alter the equilibrium of the reaction and prevent intermolecular interactions between CMC chains.^29–31^ A new nanocomposite material has also been fabricated using a CMC amide derivative (A-PH/CMC) in the presence of MWCNTs. The fabricated material was additionally investigated against the adsorption and removal performance of crystal violet and brilliant green dyes from aqueous solutions. Optimisation procedures have been studied in detail.

## Experimental

### Reagents and chemicals

CMC and 4-aminophenazone were obtained from BDH Chemicals, Ltd, Poole, England. Methanol was obtained from Aldrich Chemical Co., Ltd, Milwaukee and WC, USA. Sulphuric acid was obtained from Aldrich Chemical Co., Ltd, Milwaukee, WC, USA. The MWCNTs were purchased from the Nano-Tech Company in Egypt. The distilled water used has a resistivity of 18.2 MΩ cm. A stock solution of CV and BG dye (100 μg mL^−1^) (obtained from Aldrich Chemical Co., Ltd, Milwaukee, WC., USA) was prepared. More diluted standard (5–25 μg mL^−1^) solutions were prepared by diluting the stock solution with deionised water. A series of Britton–Robinson (BR) buffers of pH (2–11) and HCl, NaOH or both (0.1 mol L^−1^) was used as an extraction medium in the sorption process of dyes by solid phase (A-PH-CMC/MWCNT). Tap water from a chemical laboratory and sewage water were obtained from King Abdulaziz University. Seawater was obtained from the Red Sea in Saudi Arabia. The solvents and chemicals used were of analytical reagent class, and were utilised without purification.

### Instrumental characterisation

Scanning electron microscopy (SEM) images were obtained from a field emission SEM (Zeiss, Germany). The X-ray diffractometer used Ni-filtered Cu Kα radiation (FTIR Spectrometer). A Fourier transform infrared spectrometer from Thermo Fisher Scientific Company with a range of 4000–500 cm^−1^ was used for the FTIR study. An Orion pH metre (model EA 940) was used to prepare the standard solution for the experiment. A digital sensitive balance ADP 110 L with three decimal numbers was used to measure the weight of reagents and products. A hotplate 16 × 16 cm, Digital SD160 from Stuart equipment company was used. Deionised water from the Milli-Q Plus system (Millipore, Bedford, MA, USA) was used to prepare the solutions. The round bottom flasks (250 mL), condenser, conical flask, series of Britton–Robinson (BR) buffer of pH (2–11) and HCl (0.1 mol L^−1^), and shaker plate were obtained from the Thomas Xmetry Company.

### Chemical modification of CMC, CMC-H, A-PH-CMC and A-PH-CMC/MWCNT composite material

#### Acidification of Na-CMC

A beaker contained 500 mL of the following solution: 1 g Na-CMC dissolved in 100 mL of 80% ethyl alcohol solution and 10 mL of HCl. The contents of the beaker were stirred at a low speed and kept at room temperature (around 27 °C) for 30 min. Then, ethanol was used to neutralise the solution after the reaction mixture was filtered.^32^ After a series of acetone washes and an overnight drying period at 60 °C, the purified white powder was collected.

#### CMC methyl ester preparation

The acidic form of H-CMC (1 g) was suspended in 200 mL of methanol containing 1 mL of sulphuric acid. The reaction was conducted under heterogeneous conditions with stirring at 60 °C for 72 h. The reaction product (2) was filtered, washed with ethanol and acetone and dried on air.^33^

#### Amidated A-PH-CMC preparation

**Scheme 1:**
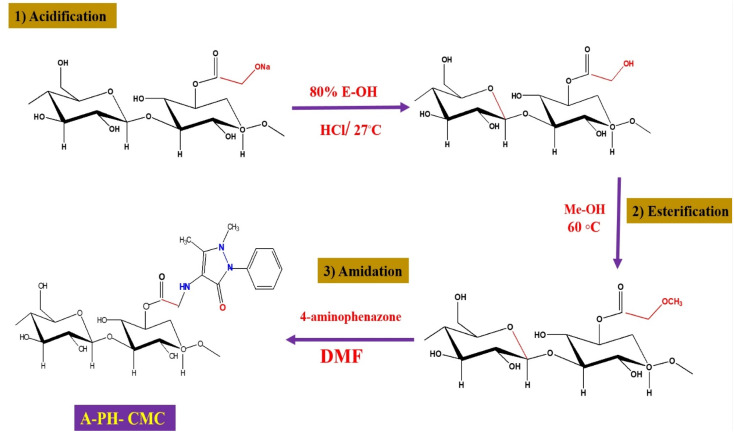
Mechanism of acidification of Na-CMC (1). The methyl ester of CMC (2). Amidated AMPH-CMC (3).

Methyl-esterified CMC produced aminophenazone-of-CMC by reacting it with the main aminophenazone amine. At this point, the mixture was stirred for 30 min. Later, the addition of the amidation reagent (10 mL) was conducted, resulting in the suspension of CMC 2 (0.5 g) in a solution of 50 mL of DMF. For the duration of the experiment, the temperature was maintained at 25 °C. The products were precipitated by acetone in distilled water. The particles were first separated, washed with an acidified combination of alcohol and dried in the open air at 55 °C for 30 min to remove any impurities. (Scheme 1)

#### Composite material formation of A-PH-CMC/MWCNTs

The material characteristics of nanostructures differ from the bulk owing to the high surface area over volume ratio and the manifestation of quantum phenomena at the nanoscale. We included five conical flakes with a volume of 200 mL inside the ultrasound machine and 20 mL of pure ethanol to conduct the process of overlapping biopolymer and nanomaterials. Afterwards, we included different MWCNT percentages (*e.g.*, 0, 5, 10, 15 and 20%) and ensured its dispersion inside the solution by keeping it for 20 min inside the ultrasound machine. We also included a conical flask with 0.5 g of the prepared substance and maintained it for 30 min inside the ultrasound device. Finally, we filtered and dried the produced sample inside a dryer for 4 h until it was ready to be used to remove the dyes from the aqueous media.

#### Environmental applications and sample collection

Water samples from the Red Sea, wastewater and tap water were used to assess the efficiency of A-PH-CMC/MWCNT nanocomposite sorbents for dye extraction. Water from the area of the Red Sea in front of Jeddah, Saudi Arabia, was used in the project as a representative sample of wastewater collected at the King Abdulaziz University treatment facility in Jeddah, Saudi Arabia. A representative wastewater sample was collected at the King Abdulaziz University treatment facility in Jeddah, Saudi Arabia. The samples were filtered through a 0.45 μm membrane filter, and kept in Teflon bottles at 5 °C in the dark. A 100 mL sample was adjusted to pH 8.0 for the CV dye, and adjusted to pH 6.0 for the BG dye. It was then passed through the solid phase A-PH-CMC/MWCNT nanocomposites. The recovered dyes were calculated spectrophotometrically.

## Results and discussion

### Characterisation studies

**Fig. 1:**
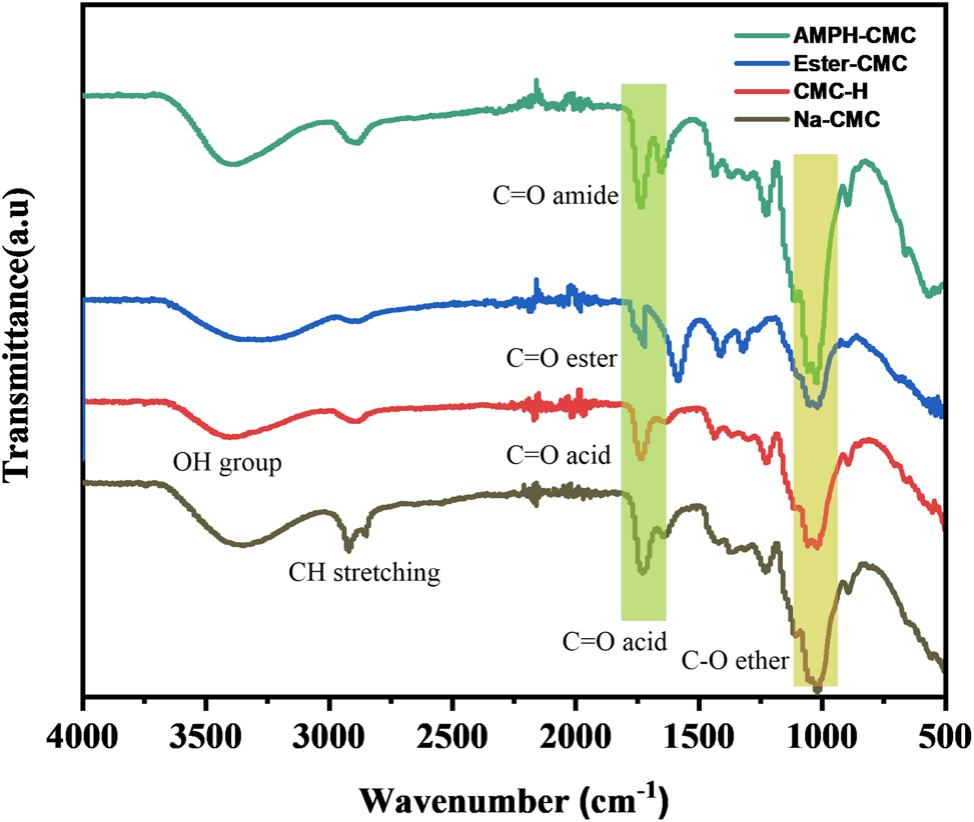
FTIR spectra of pure Na-CMC, H-CMC, ester-CMC, and A-PH-CMCMWCNTs.

The IR spectra of CMC polymers in Fig. 1 show the primary bands and function groups. There was a single bond OH stretching in the FT-IR spectrum of CMC at 3400 cm^−1^, hydrocarbon stretching (C single bond H stretching of single bond CH_2_ groups) at 2860 cm^−1^, a carbonyl stretching (C double bond O stretching) at 1620 cm^−1^, a single bond CH_2_ scissors at 1420 cm^−1^, and ether stretching (single bond O stretching) at 1030 cm^−1^, as previously observed.^34,35^ H-CMC causes a new signal at 1720 cm^−1^, the C=O stretching vibration of the generated carboxylic acid groups, indicating that acidification was successful. Adsorbed water molecules might have been responsible for the 1630 cm^−1^ shoulder.^36^ After the esterification reaction between methanol and CMC, in which the carboxylic group and methanol were bonded after removing water molecules, the ester-CMC compound was produced. A new curve at 1310 cm^−1^ representing the ester group was observed.^37,38^ A-PH-CMC/MWCNTs cause the aromatic ring of A-PH-CMC to have C=C stretching vibrations between 1440 and 1503 cm^−1^, and the covalent coupling with the amine was proven by the appearance of two additional intense bands at about 1662 and 1551 cm^−1^ attributable to the amide. Moreover, in the A-PH-CMC/MWCNT nanocomposites, absorptions at 2950 cm^−1^ and 2850 cm^−1^ were related to C–H stretch modes. At the same time, the peak observed at 1640 cm^−1^ corresponded to the C–C stretch of the MWCNTs, and the peak at 1460 cm^−1^ was due to the C–H bending mode of the alkyl chain^39–41^.

#### A-PH-CMC/MWCNT nanocomposites

**Fig. 2:**
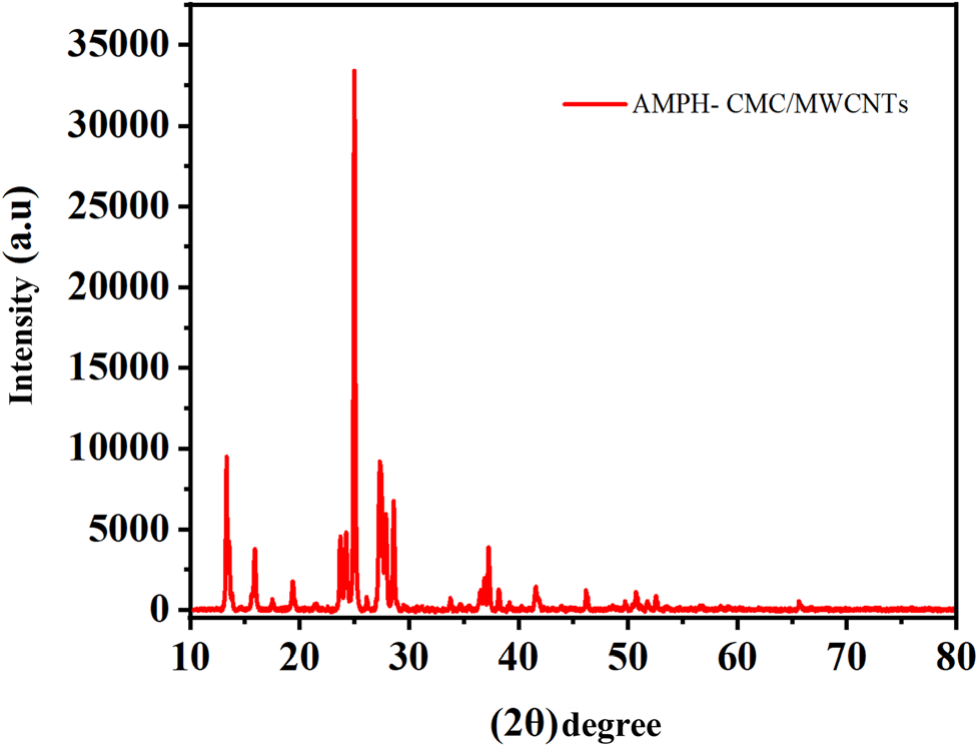
XRD pattern of neat A-PH-CMC/MWCNT nanocomposites.

An X-ray diffraction pattern (Fig. 2) was conducted to investigate the crystal lattice structure of the A-PH-CMC/MWCNT adsorbent materials. The amorphous carboxymethyl cellulose polymer crystals peaked at around 2*θ* = 21.32°.^42^ The MWCNT network was responsible for a diffraction peak at 26°,^43^ illustrating the crystalline structure of the prepared material, as seen in the diffraction pattern. Its view increased at 2*θ* = 25.55°, 43.45°, 53.27° and 53.91°, which were indexed to 111, 010, 222 and 112, respectively. Each corresponds to a particular card in the graphite structure (JCPDS card No. 75-2078). This set of peaks was consistent with those observed in MWCNTs.^44^

**Fig. 3:**
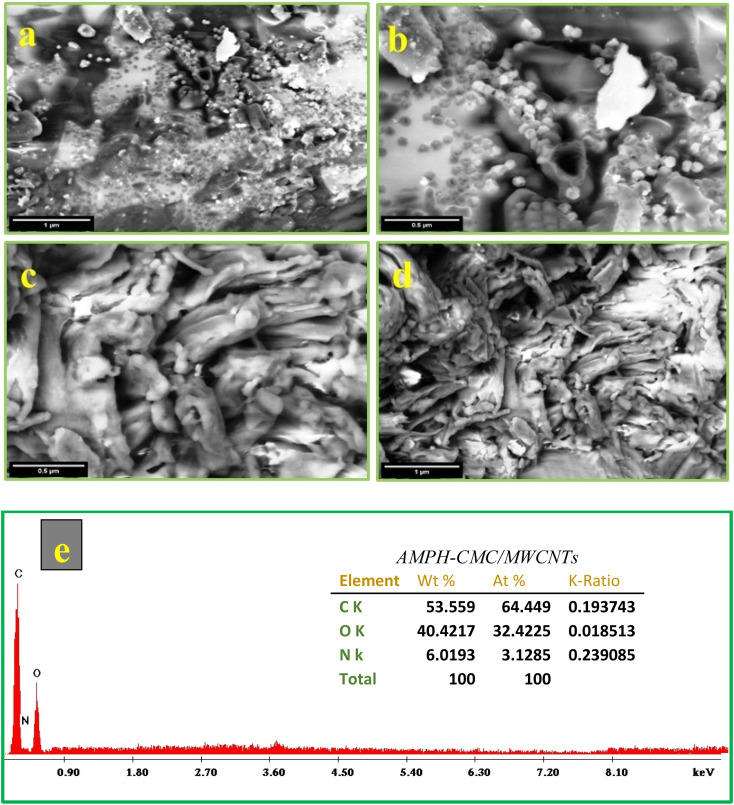
SEM micrographs at different magnifications. (a and b) A-PH-CMC, (c and d) A-PH-CMC/MWCNTs nanocomposite, and (e) EDX spectrum of the A-PH-CMC/MWCNT nanocomposites.

The surface morphology of the A-PH-CMC and A-PH-CMC/MWCNT nanocomposites was conducted using the SEM instrument. The obtained images are illustrated in Fig. 3. A-PH-CMC images (Fig. 3a and b) show a significant change to pure CMC due to the chemical modification that occurs. A smooth surface with immersed globular particles and tiny pores was shown by the images. Furthermore, the embedded intensity of the MWCNTs into the modified CMC sample with suitable compatibility was shown by the A-PH-CMC/MWCNT nanocomposite images (Fig. 3c and d). Accumulated MWCNTs appeared due to the higher percentage of nanotube loading in the polymer matrix. The EDX analysis for A-PH-CMC/MWCNT nanocomposites is shown in Fig. 3e. Following the proposed structure, peaks corresponding to the C, O and N elements were observed for the measured sample. The spectrum indicates the presence of C, O and N elements, together with their corresponding binding energies of 0.25, 0.35 and 0.48 keV, respectively. No additional peaks were determined.

### Environmental treatments

#### The extraction process using batches

Regarding the actual weight (0.01 ± 0.002 g) of the A-PH-CMC/MWCNT nanocomposite, the solid phase was balanced using a water-based solution (50 mL) of CV (5 mg L^−1^) at a pH of 8.0 and (0.01 ± 0.002 g) of the A-PH-CMC/MWCNT nanocomposites. The solid phase was balanced using a water-based solution (50 mL) consisting of BG dye (5 mg L^−1^) at a pH of 6.0. The sample solutions were shaken for 75 min on a mechanical shaker. The aqueous layer was broken up, and the number of dyes remaining in the aqueous phase was determined photometrically.^45^ The number of dyes kept on the A-PH-CMC/MWCNT nanocomposite solid phase was counted from the difference in absorbance between dyes in the aqueous phase before (*A*_b_) and after (*A*_f_) being taken out. The amount of sorption (%*E*), number of dyes kept at equilibrium (*q*_e_) per unit mass of solid sorbent (mol g^−1^) and the distribution coefficient (*K*_d_) of sorbed analyte onto the A-PH-CMC/MWCNT nanocomposites were computed as described. The %*E* and *K*_*d*_ values are the averages of three separate measurements, with a typical precision of 2%. Following these procedures, the influence of shaking time and temperature on the retention of dyes by the A-PH-CMC/MWCNT nanocomposite sorbents was studied.

#### Adsorption study

**Fig. 4:**
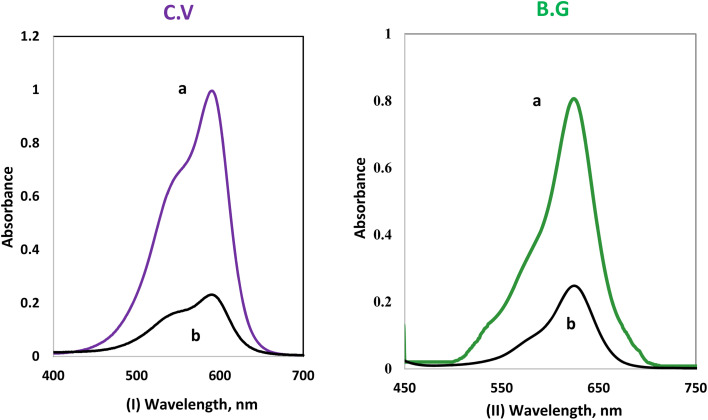
The difference between the electronic spectra of the (I) CV dye and (II) BG dye in the aqueous phase without the solid phase (a), and after the addition of the solid phase (A-PH-CMC/MWCNTs) after 75 min of shaking (b).

It was shown that the electronic spectrum of BG and CV recorded in the form of an aqueous solution had an absorption peak at 691 ± 3 nm for CV and 626 ± 3 nm for BG. However, these peaks decreased dramatically after shaking with solid phase A-PH-CMC/MWCNT nanocomposites. Such behaviour confirms the efficiency of the A-PH-CMC/MWCNT nanocomposites for the removal of dyes from aqueous solution, as illustrated in Fig. 4.

#### CV and BG dye retention e1 profile on A-PH-CMC/MWCNTs from aqueous solution

**Fig. 5:**
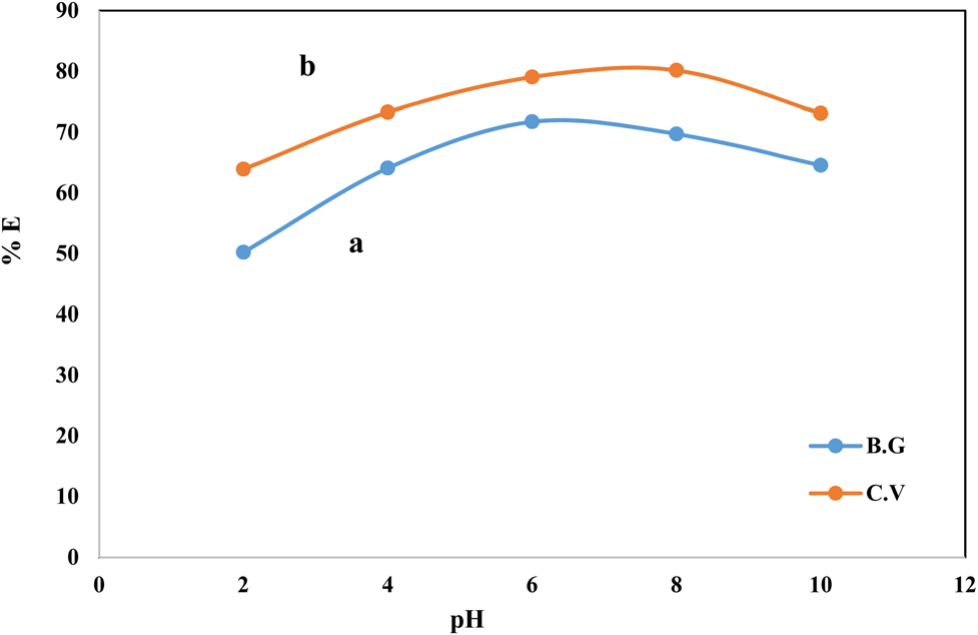
Effect of the solution pH on the sorption percentage of BG (a) and CV (b) dyes from aqueous solutions onto (0.01 ± 0.002 g) A-PH-CMC/MWCNT nanocomposites with shaking time 75 min at 25 ± 0.1 °C.

The solution pH is one of the critical parameters for achieving adsorption and recovery for heavy metal ions and dyes. The sorption profile of the aqueous solutions containing CV and BG dyes at different pH values by the A-PH-CMC/MWCNT nanocomposites solid phase was critically studied after shaking for 75 min at room temperature. The number of dyes in the aqueous phase after equilibrium was determined photometrically.^43^ The sorption percentage (%*E*) of CV sorption onto the A-PH-CMC/MWCNT increased markedly upon increasing the solution pH until a value of 8.0 was reached. After this, the percentage decreased with increasing pH. The sorption percentage %*E* of BG sorption onto the A-PH-CMC/MWCNT nanocomposites increased markedly on increasing the solution pH until its value reached 6.0. After this, the percentage decreased with increasing pH. Representative data are shown in Fig. 5. Therefore, the pH value was adjusted using HCl or NaOH until the optimum value of pH 8.0 was obtained for CV. A pH of 6.0 was selected as the optimum value for BG in the subsequent analysis.

**Fig. 6:**
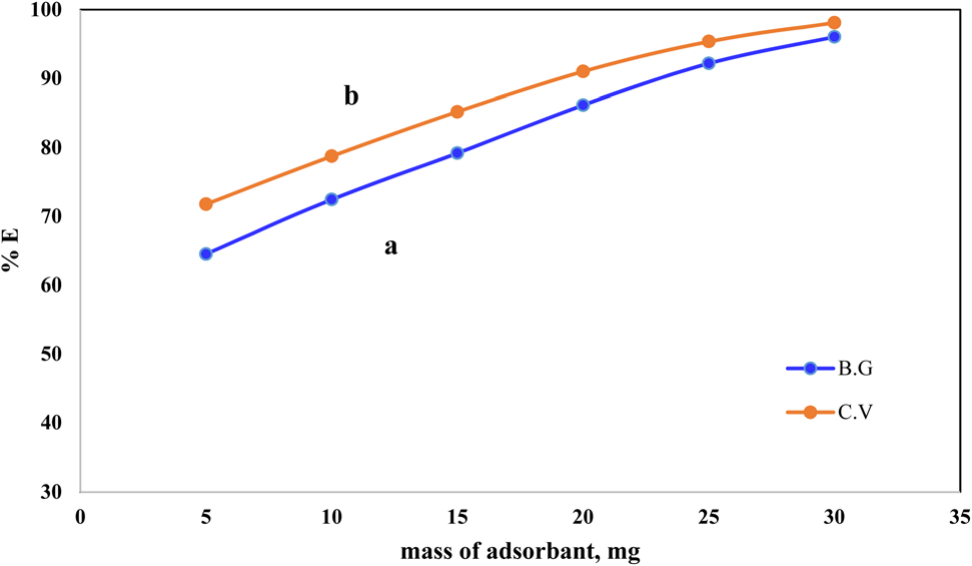
Effect of A-PH-CMC/MWCNT nanocomposites mass on the adsorption percentage of dyes species BG (a) and CV (b) from aqueous solutions with a shaking time of 75 min and at a temperature of 25 °C.

The effect of the solid phase A-PH-CMC/MWCNT mass on the percentage of dyes adsorbed from the aqueous solution was studied with CV and BG at a concentration of 5 mg L^−1^ (Fig. 6). The dye percentage removed from the aqueous solution increased from 71% to 98% as the A-PH-CMC/MWCNT nanocomposites dose increased from 5 mg to 30 mg for the CV dye. At the same time, it increased from 64% to 96% with the increase in the A-PH-CMC/MWCNT nanocomposites dose, from 5 mg to 30 mg for the BG dye. The proportion of adsorbed molecules rose due to more adsorption-active sites because of the higher solid-phase concentration. Hence, 10 mg of A-PH-CMC/MWCNT nanocomposites, which corresponded to 72% for BG and 78% for CV, was used to observe the effect of the other parameters on the adsorption process.

**Fig. 7:**
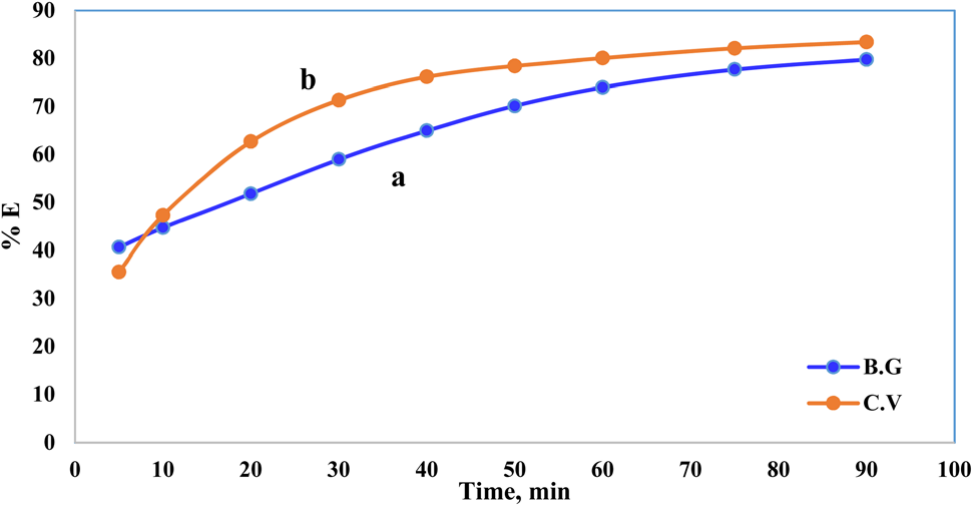
Effect of shaking time on the sorption percentage of dye species BG (a) and CV (b) from aqueous solutions onto A-PH-CMC/MWCNT nanocomposites at a temperature of 25 °C.

**Fig. 8:**
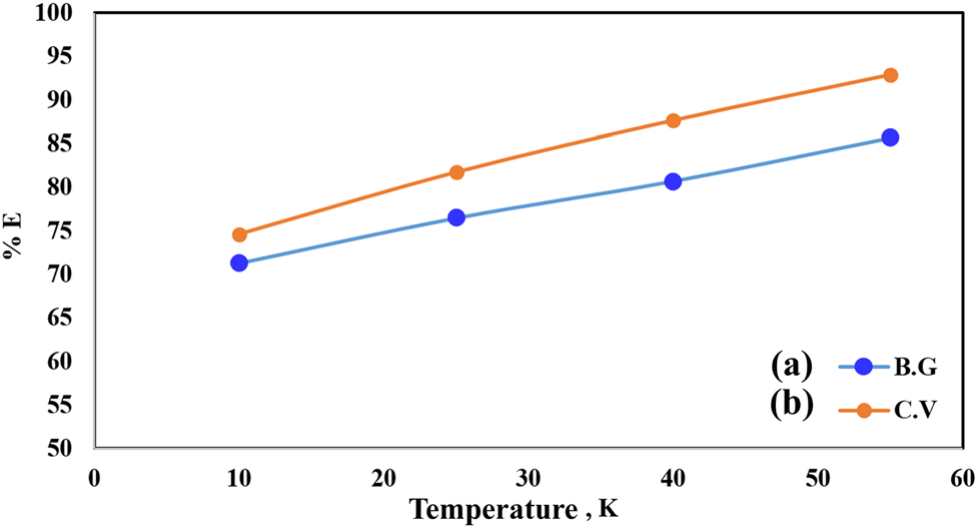
Effect of temperature on the sorption percentage of dye species BG (a) and CV (b) from aqueous solutions onto A-PH-CMC/MWCNT nanocomposites at 283 K, 298 K, 313 K and 328 K.

Pollutant removal by adsorption depends on several factors, but the contact duration between the adsorbate and adsorbent is the most important. Dye removal efficiency by A-PH-CMC/MWCNT nanocomposites was investigated as a function of contact time. The findings are shown in Fig. 7, in which the adsorption process is accelerated with increasing contact time. Most dyes were adsorbed in the first 50 min, when this impact was most pronounced. Within 75 min, the dye clearance percentage stabilised. This suggests that two distinct processes were involved in the adsorption of dyes on the A-PH-CMC/MWCNT nanocomposites, with the first being the most rapid and consisting of the transfer of dyes species from the aqueous phase to the exterior surface of A-PH-CMC/MWCNTs. The second was the slower step, which was the diffusion of dye species between the A-PH-CMC/MWCNT nanocomposite bundles. The study also examined how the increasing temperature of the solution affected the adsorption process. Results were compared at a consistent shaking time over four temperatures (283 K, 298 K, 313 K and 328 K). When the solution temperature was raised from 283 K to 298 K, 313 K and 328 K, a statistically significant increase in the proportion of dyes removed by A-PH-CMC/MWCNT nanocomposites was observed (Fig. 8). According to these findings, this adsorption mechanism was endothermic.

**Fig. 9:**
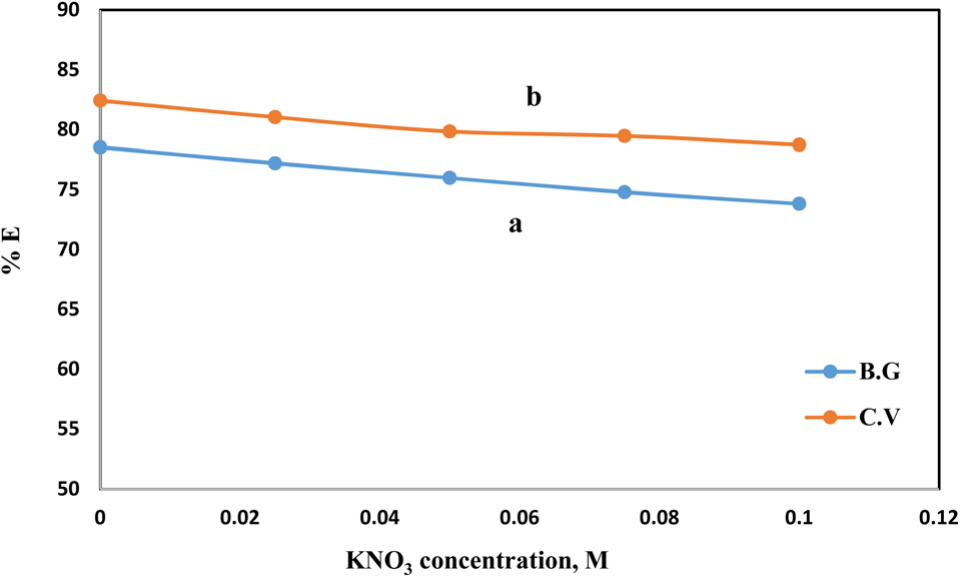
Effect of KNO_3_ concentration on the adsorption percentage of dyes species BG (a) and CV (b) from aqueous solutions onto A-PH-CMC/MWCNT nanocomposites at a temperature of 25 °C.

Once distinct adsorption scenarios may be induced by ionic strength, in which electrostatic interactions between the solid phase surfaces and the dye species are either attractive or repulsive, it is of paramount importance. The adsorption of BG and CV dyes onto the A-PH-CMC/MWCNT nanocomposites solid phase was studied concerning the ionic strength available. Adsorption experiments were performed with varying doses of KNO_3_ (*i.e.*, 0.025, 0.05, 0.075 and 0.1 mol L^−1^) to alter the ionic strength (Fig. 9). Increasing the ionic strength of the aqueous solution resulted in a marginal reduction in the proportion of dyes species absorbed. This may be explained by the fact that the presence of a cation, such as K^+^, reduces the interaction of the dye species with the adsorbent surface. This happens because of the build-up of charge around the adsorbent surface.^39^

#### Kinetic behaviour of BG and CV dye sorption onto A-PH-CMC/MWCNT nanocomposites

It is crucial to understand the sorption kinetics of contaminants, such as dye species, from aqueous solutions because they shed light on the reaction routes and the mechanisms underlying the sorption processes. Film diffusion and intraparticle diffusion have a role in the retention of dye species on solid phase A-PH-CMC/MWCNT nanocomposites. The faster of the two will determine the transport rate. The computation of the half-life time *t*(1/2) of dye sorption from the aqueous solutions onto the solid sorbents A-PH-CMC/MWCNT nanocomposites provided evidence in favour of the conclusion drawn from the effect of shaking time. The plots of log *C*/*C*_o_*vs.* time for the sorption of BG and CV dyes onto A-PH-CMC/MWCNT nanocomposites were used to obtain the values of *t*1/2. According to earlier reports, the value of *t*(1/2) was determined to be 1.5 ± 0.04 min for BG and 1.6 ± 0.05 min for CV dyes.^46^ Therefore, the overall transport rate is controlled by whichever of the film and intraparticle diffusion processes is faster in the case of dye species sorption onto the A-PH-CMC/MWCNT nanocomposite sorbent. The Weber–Morris model was applied to the dye species absorbed by the A-PH-CMC/MWCNT sorbent:^47^

**Fig. 10:**
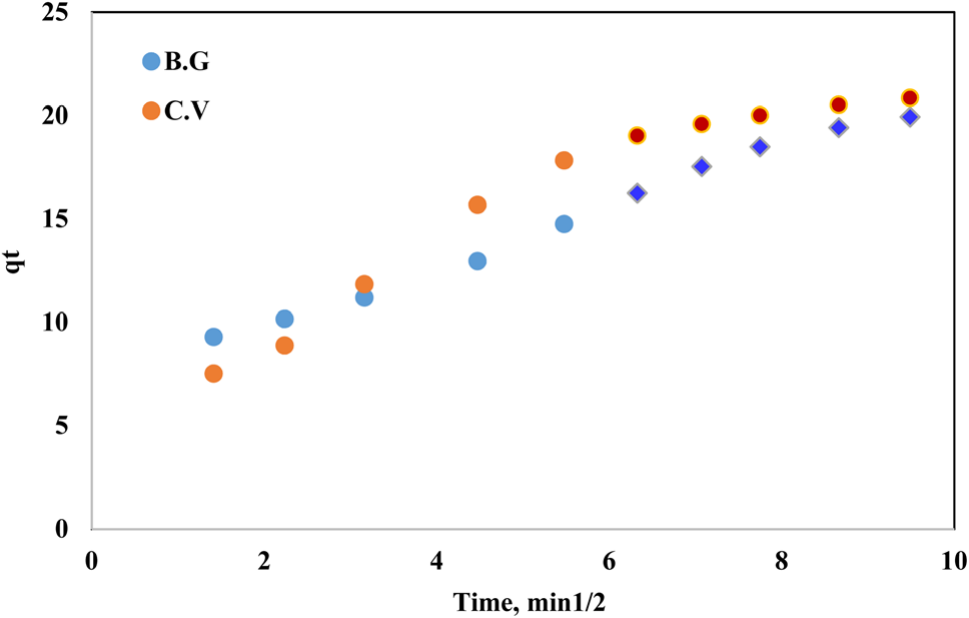
Weber–Morris plot of the sorbed dyes BG and CV from aqueous solutions onto the A-PH-CMC/MWCNT nanocomposites *versus* the square root of time. Experimental conditions are mentioned in the batch extraction step.

1 *q*_*t*_ = *R*_d_(*t*)1/2

where *R*_d_ is the intraparticle transport rate constant, and *q*_*t*_ is the concentration of sorbed dye at time *t*. The plot of *q*_*t*_*versus* time is shown in Fig. 10. By calculating *R*_d_ from the separate slopes of Weber–Morris plots (Fig. 7), we found that the BG dye had an *R*_d_ value of 1.43 mg g^−1^ and an *R*^2^ value of 0.991, while the CV dye had an *R*_d_ value of 1.75 mg g^−1^ and an *R*^2^ value of 0.919.

The fractional power function kinetic model was modified and derived from the Freundlich equation. This was represented by the following formula:^48^

**Fig. 11:**
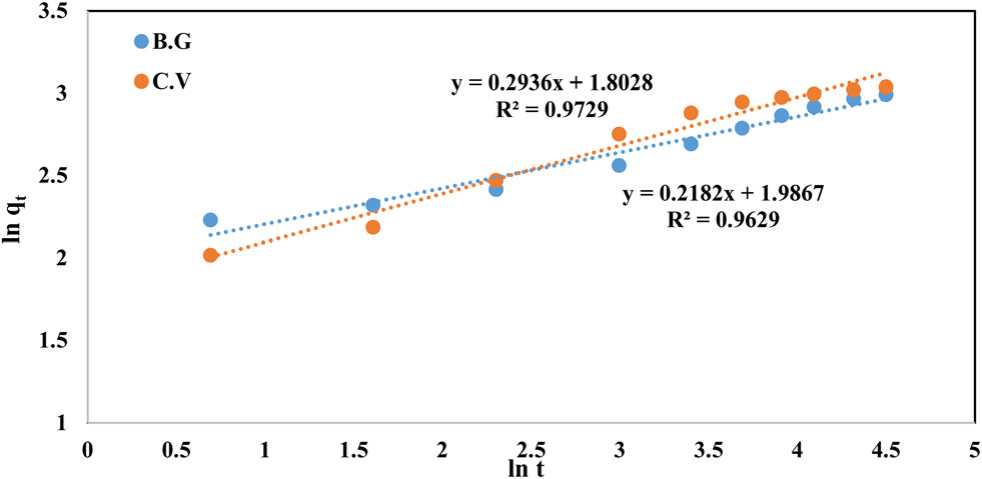
Fractional power model plots of dyes BG and CV from aqueous solutions onto A-PH-CMC/MWCNT nanocomposites. Experimental conditions are mentioned in the batch extraction.

2 ln *q*_*t*_ = ln *a* + *b* ln *t*

where *q*_*t*_ (mg g^−1^) is the amount of dye species (BG and CV) adsorbed per unit mass of A-PH-CMC/MWCNT nanocomposites at any time *t*. At the same time, *a* and *b* are coefficients with *b* equal to 1. When the fractional power function equation was applied to the experimental data of the adsorption process, as shown in Fig. 11, the data fit well, with a correlation coefficient (*R*^2^) value of 0.963 for the BG dyes and 0.973 for the CV dyes. The values of *a* and *b* are provided in Table 1. These findings may indicate that the fractional power function kinetic model is insufficient for describing the dye species adsorption by the A-PH-CMC/MWCNT nanocomposite.

Adsorption of BG and CV dyes onto A-PH-CMC/MWCNT nanocomposites at 293 K, using varying kinetic model parameters

**Table:** 

Weber–Morris model				
		*R*_d_		*R* ^2^
BG		1.43		0.991
CV		1.75		0.919

**Table:** 

Fractional power function kinetic model				
	*a*	*b*	*ab*	*R* ^2^
BG	7.24	0.218	1.57	0.963
CV	6.05	0.293	1.77	0.973

**Table:** 

The pseudo-first-order kinetic (Lagregen) model				
	*q*_e_, exp (mg g^−1^)	*q*_e_, calc (mg g^−1^)	*k*_1_	R^2^
BG	19.93	13.8	0.04	0.958
CV	20.85	14.4	0.05	0.995

**Table:** 

The pseudo-second-order kinetic model				
	*q*_e_, exp (mg g^−1^)	*q*_e_, calc (mg g^−1^)	*k*_2_	R^2^
BG	19.93	20.83	5.9 × 10^−3^	0.991
CV	20.85	22.42	6.2 × 10^−3^	0.998

**Table:** 

Elovich kinetic model				
	*α*, (g mg^−1^ min^−1^)	*β*, (mg g^−1^ min^−1^)		*R* ^2^
BG	2.05	3.015		0.926
CV	0.655	3.944		0.976

Correlation-coefficient values for the fractional power function, the Lagergren pseudo-first order, the pseudo-second order and the Elovich models used to fit the experimental data on the adsorption of dyes species (BG and CV) are shown in Table 1. The pseudo-second-order kinetic model was the most appropriate to describe the adsorption of dyes species by the A-PH-CMC/MWCNT nanocomposites.

When modelling adsorption rates in liquid-phase systems, the Lagergren equation is often used. The Lagergren equation was applied to the data describing the shift in sorption of dyes (BG and CV) from aqueous media onto the A-PH-CMC/MWCNT nanocomposites solid phase:^49^

**Fig. 12:**
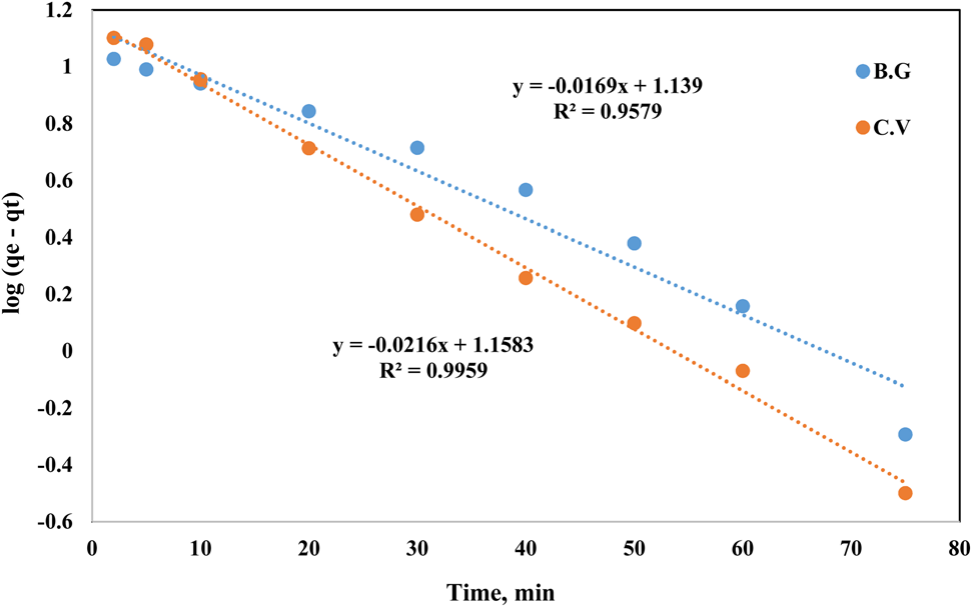
Lagergren plot of dyes BG and CV uptake onto A-PH-CMC/MWCNT nanocomposites *versus* time. Experimental conditions are mentioned in the batch extraction step.

3 log(*q*_e_ − *q*_*t*_) = log *q*_e_ − *K* Lager*t*/2.303

where *q*_e_ is the equilibrium sorbed quantity of chemical species per unit mass of sorbent, *K* Lager is the first-order overall rate constant for the retention process and *t* is the duration. A linear relationship between log(*q*_e_ − *q*_*t*_) and time is shown in Fig. 12. The calculated values of *K* Lager and *q*_e_ for the BG dye were 0.040 min^−1^ and 13.8 mg g^−1^, respectively, with a correlation coefficient (*R*^2^) of 0.958. For the CV dye, these values were 0.050 min^−1^ and 14.4 mg g^−1^, respectively, with a correlation coefficient (*R*^2^) of 0.995.^32^

The pseudo-second-order equation has also been interpreted as a special kind of Langmuir kinetics,^42^ assuming that (i) the adsorbate concentration is constant in time and (ii) the total number of binding sites depends on the amount of adsorbate adsorbed at equilibrium. The linearised form of the pseudo-second-order rate was expressed by the following equation:4
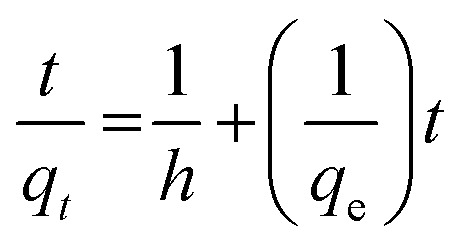


**Fig. 13:**
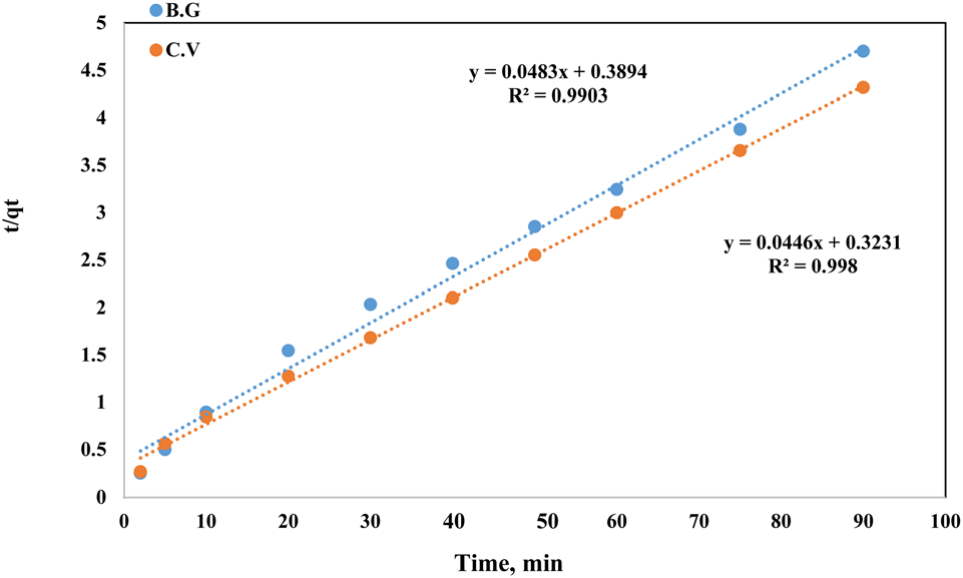
Pseudo-second order plot of dyes BG and CV uptake onto A-PH-CMC/MWCNT nanocomposites *versus* time. Experimental conditions are mentioned in the batch extraction step.

In this equation, *q*_e_ and *q*_*t*_ are the equilibrium and time-independent quantities adsorbed per unit mass, respectively, while *h* is the initial sorption rate. *T*/*q*_*t*_*vs.* time graphs were linear under these conditions, as seen in Fig. 13. An excellent correlation (*R*^2^ = 0.991) was discovered between the slope and intercept for BG dye and the second-order rate constant (*k*_2_) and equilibrium capacity (*q*_e_) for dye species. These values were determined to be equivalent at 5.9 × 10^−3^ g (mg min)^−1^ and 20.83 mg g^−1^. An excellent correlation (*R*^2^ = 0.998) was discovered between the concentrations of CV, the second-order rate constant (*k*_2_) and equilibrium capacity (*q*_e_) for dyes species. These values were determined to be equivalent at 6.2 × 10^−3^ g (mg min)^−1^ 22.42 mg g^−1^. Dyes and their rates of consumption (in mg min^−1^) and concentrations (mg g^−1^) were determined to be identical. All of the experimental results matched well with the values and the values of the pseudo-second-order rate constant, *k*_2_, which frequently relied on observed variables, such as those starting metal concentration, solution pH and temperature.

The rate equation was derived from the Elovich model of adsorption capacity.^28^ This model works well for systems with heterogeneous adsorptive surfaces, and may be used for chemisorption kinetics in general. This model is described by the following formula:

5 *q*_*t*_ = *β* ln(*αβ*) + *β* ln *t*

**Fig. 14:**
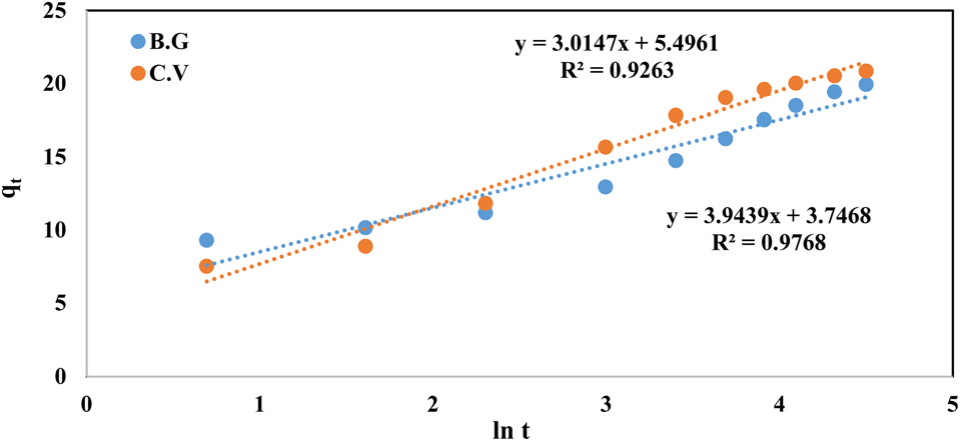
Elovich model plot for dyes BG and CV uptake onto A-PH-CMC/MWCNT nanocomposites *versus* time. Experimental conditions are mentioned in the batch extraction step.

The initial adsorption rate is denoted by (α), while the desorption coefficient is represented by (*β*). Linearity was observed in the plot of *q*_*t*_ against the time in Fig. 14. For the BG dye adsorbed onto A-PH-CMC/MWCNTs, the Elovich values were equal to 2.05 g mg^−1^ min^−1^ and 3.015 mg g^−1^ min^−1^, respectively (*R*^2^ = 0.926). At the same time, the dye adsorbed onto the A-PH-CMC/MWCNT nanocomposites for CV was equivalent to 0.655 g mg^−1^.

Thermodynamic characteristics of BG and CV dye retention onto A-PH-CMC/MWCNT nanocomposites dye species retention onto the A-PH-CMC/MWCNT nanocomposites was investigated by studying the sorption of BG and CV dyes onto the solid phase at different temperatures (283–328 K). The following equations were used to determine values for the thermodynamic constants (Δ*H*, Δ*S* and Δ*G*):^39^6
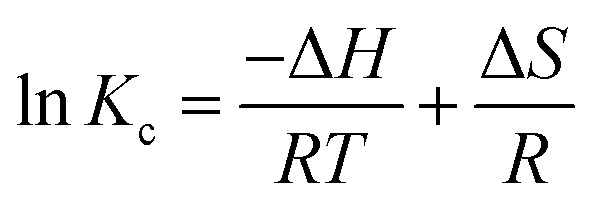

7 Δ*G* = Δ*H* − *T*Δ*S*

**Fig. 15:**
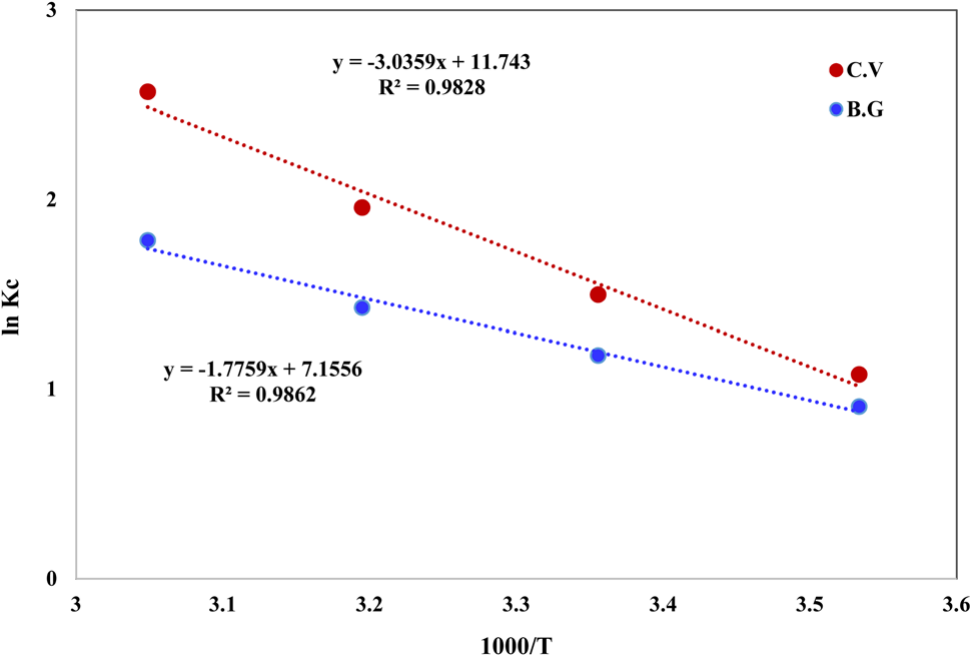
Plot of ln KC of BG and CV dyes sorption from aqueous media onto A-PH-CMC/MWCNT nanocomposites *versus* 1000/*T*.

8 Δ*G* = −*RT* ln *K*_c_

where Δ*H*, Δ*G* and Δ*S* represent the changes in enthalpy, Gibbs free energy and entropy, respectively. *K* is the equilibrium constant, *T* is the temperature in Kelvin, and *R* is the gas constant (8.314 J K^−1^ mol^−1^). Using this equation, we were able to determine the *K*_C_ values for the equilibrium retention of BG and CV dyes from the aqueous test solution onto the solid:9
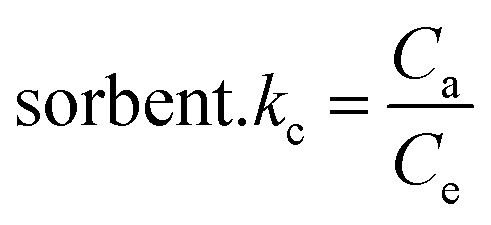
where *C*_a_ is the quantity of BG and CV dyes adsorbed onto the solid phase per litre at equilibrium, and *C*_e_ is the equilibrium concentration of BG and CV dyes in an aqueous solution (in millimoles per litre) (mg L^−1^). The retention of BG and CV dyes onto A-PH-CMC/MWCNT nanocomposites solid phase was linear, as shown by a plot of ln *K*_C_*vs.* 1000/*T* (Fig. 15) across the temperature range (283–328 K). The retention process of BG and CV dyes onto the utilised sorbents is endothermic, as shown by the fact that the equilibrium constant rises with increasing temperature. The numerical values of Δ*H*, Δ*S* and Δ*G* for dyes species (BG and CV) retention calculated from the slope and intercept of the linear plot of ln *K*_C_*versus* 1000/*T* (Fig. 15) were 14.7 ± 0.2 kJ mol^−1^, 59.5 ± 0.4 J mol^−1^ K^−1^ and −3.0 ± 0.1 kJ mol^−1^ (at 298 K), respectively, for the BG dye. They were 25.2 ± 0.3 kJ mol^−1^, 97.6 ± 0.5 J mol^−1^ K^−1^ and −3.9 ± 0.2 kJ mol^−1^ (at 298 K), respectively, for the BG dye.

The extent to which the absorption process is endothermic may be inferred from the *H* value, which indicates the difference in bond energy between the sorbent and the analyte. The release of water molecules from the hydration sphere during the adsorption processes resulted in a positive value of *S* for the solid phase A-PH-CMC/MWCNT nanocomposites. This is an indication of an increase in the degree of freedom at the solid–liquid interface, which is commonly observed during the binding of dyes species (BG and CV). The generated solid phase had a negative *G* at 298 K, suggesting that the dye species were retained onto A-PH-CMC/MWCNT nanocomposites by a spontaneous and physical sorption process.

### Environmental applications

**Fig. 16:**
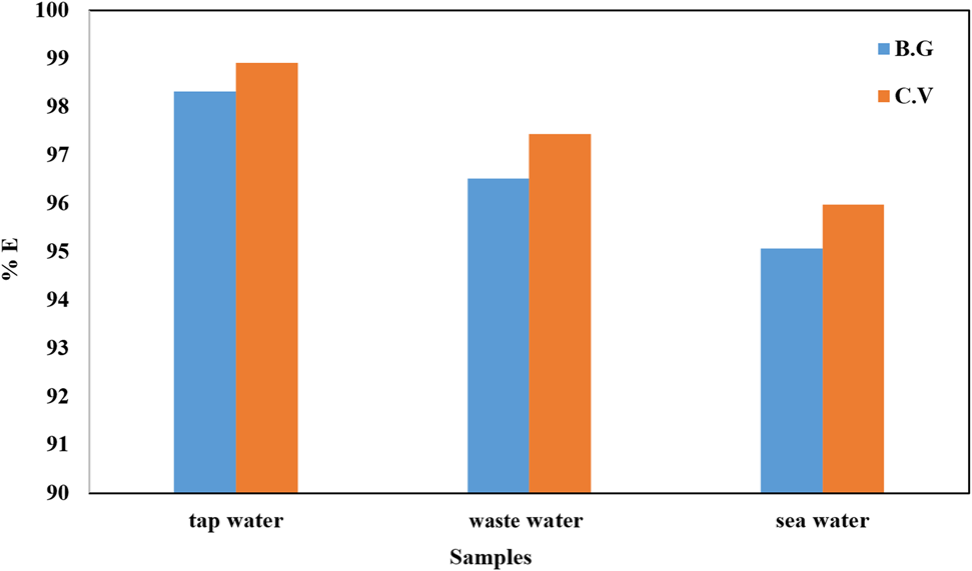
The removal percentages of BG and CV dyes from different actual samples by the formed A-PH-CMC/MWCNT solid phase (experimental conditions: 50 mL solution, BG concentration of 5 mg L^−1^, a pH of 6.0 and 35 mg of A-PH-CMC/MWCNT nanocomposites for BG dye and 50 ml solution, a pH of 8.0, CV concentration 5 mg L^−1^ and 35 mg of A-PH-CMC/MWCNTs for CV dye and shaking time of 90 min and a temperature of 298 K).

Actual environmental samples must be studied to observe whether the A-PH-CMC/MWCNT nanocomposite may be used to remove estrogenic chemicals. Water samples from the Red Sea, in front of Jeddah City, KSA, wastewater from the wastewater treatment plant at King Abdulaziz University, Jeddah City, KSA and tap water from the Chemistry Department labs at King Abdulaziz University, Jeddah City, KSA, were used in this experiment. The three observed concentrations of the dye species samples (BG and CV) were below the detection threshold of the UV-vis measurement. After correcting the pH to 6.0 and stirring the solution for 90 min at a temperature of 298 K, 5 mg L^−1^ of BG dye was added to each of the three samples. Then, 35 mg A-PH-CMC/MWCNT solid phase was added. Three samples were spiked with 5 mg L^−1^ of CV dye, and 35 mg A-PH-CMC/MWCNT nanocomposites solid phase was added to the solution after correcting the pH to 8.0 and shaking the solution for 90 min at a temperature of 298 K. Dye removal rates for BG and CV are shown as a percentage of the total for the actual samples in Fig. 16. Nearly the same removal percentage was achieved for four cycles. The measurement of BG and CV dyes after the A-PH-CMC/MWCNT nanocomposites were collected, washed, dried and reused. (Table 2)

**Table:** The compared adsorption capabilities and adsorption times of dyes in equilibrium

Dyes	Solid phase	Shaking time	Uptake capacity	Ref.
CV	GBC700	40 min	23.71	46
CV	Coniferous pinus bark powder	120 min	32.78	47
CV	GBC300	240 min	11.02	48
CV	Nano-porous carbon from tomato waste	150 min	68.97	49
**CV**	**A-PH-CMC/MWCNT**	**50 min**	**22.42**	**This work**
BG	Halloysite nanoclay	40 min	12.5	42
BG	Carboxylated alginic acid	60 min	1.06	50
BG	Fava bean peels	80 min	28.14	51
BG	Nano hydroxyapatite/Chitosan composite	60 min	30.2	52
BG	activated carbon derived from date pits	55 min	77.8	53
**BG**	**A-PH-CMC/MWCNT**	**50 min**	**20.83**	**This work**

## Conclusion

The A-PH-CMC/MWCNT nanocomposite adsorbents produced in this study had promising adsorption performance. FESEM and EDX analysis findings demonstrated a uniformly porous surface, and the produced adsorbent showed adequate CV and BG adsorptions. The FTIR study revealed that the A-PH-CMC/MWCNTs adsorption affinity resulted from their single-layered planes bearing functional groups (hydroxyl, ester and amide). The highest adsorption quantity for BG dye was up to 5.9 × 10^−3^ g (mg min)^−1^ and 20.83 mg g^−1^. In CV, these values were 6.2 × 10^−3^ g (mg min)^−1^ and 22.42 mg g^−1^.

Meanwhile, the A-PH-CMC/MWCNT nanocomposites had adequate recyclability in the actual samples, and the resorption ratio could still approach 95%. Moreover, the pH, contact time, adsorbent dose and temperature and KNO_3_ effects, which were the most essential factors in the adsorption process, were fine-tuned. As a further step, we calculated the adsorption kinetics of both dyes using several models. Ultimately, we settled on the pseudo-second-order model as the most accurate representation of the data. Electrostatic forces were shown to play a significant role in the adsorption of the BG and CV dyes onto the A-PH-CMC/MWCNT nanocomposites, as demonstrated by the desorption tests. These results show that A-PH-CMC/MWCNT nanocomposites are the most effective low-cost adsorbents for removing harmful BG and CV dyes from wastewater treatment systems.

## Conflicts of interest

There are no conflicts to declare.

## Supplementary Material
